# Material Extrusion 3D Printing of PEEK-Based Composites

**DOI:** 10.3390/polym15163412

**Published:** 2023-08-15

**Authors:** Thomas Hanemann, Alexander Klein, Siegfried Baumgärtner, Judith Jung, David Wilhelm, Steffen Antusch

**Affiliations:** 1 Institute for Applied Materials, Karlsruhe Institute of Technology, Hermann-von-Helmholtz-Platz 1, D-76344 Eggenstein-Leopoldshafen, Germany; a.klein@kit.edu (A.K.); siegfried.baumgaertner@kit.edu (S.B.); judith.jung@kit.edu (J.J.); david.wilhelm@gmx.net (D.W.); steffen.antusch@kit.edu (S.A.); 2 Department of Microsystems Engineering, University Freiburg, Georges-Koehler-Allee 102, D-79110 Freiburg, Germany

**Keywords:** material extrusion, MEX, FFF, PEEK, PEEK-based composites, mechanical properties, thermal conductivity

## Abstract

High-performance thermoplastics like polyetheretherketone (PEEK), with their outstanding thermal stability, mechanical properties and chemical stability, have great potential for various structural applications. Combining with additive manufacturing methods extends further PEEK usage, e.g., as a mold insert material in polymer melt processing like injection molding. Mold inserts must possess a certain mechanical stability, a low surface roughness as well as a good thermal conductivity for the temperature control during the molding process. With this in mind, the commercially available high-performance thermoplastic PEEK was doped with small amounts of carbon nanotubes (CNT, 6 wt%) and copper particles (10 wt%) targeting enhanced thermomechanical properties and a higher thermal conductivity. The composites were realized by a commercial combined compounder and filament maker for the usage in a material extrusion (MEX)-based 3D-printer following the fused filament fabrication (FFF) principle. Commercial filaments made from PEEK and carbon fiber reinforced PEEK were used as reference systems. The impact of the filler and the MEX printing conditions like printing temperature, printing speed and infill orientation on the PEEK properties were characterized comprehensively by tensile testing, fracture imaging and surface roughness measurements. In addition, the thermal conductivity was determined by the laser-flash method in combination with differential scanning calorimetry and Archimedes density measurement. The addition of fillers did not alter the measured tensile strength in comparison to pure PEEK significantly. The fracture images showed a good printing quality without the MEX-typical voids between and within the deposited layers. Higher printing temperatures caused a reduction of the surface roughness and, in some cases, an enhanced ductile behavior. The thermal conductivity could be increased by the addition of the CNTs. Following the given results, the most critical process step is the compounding procedure, because for a reliable process–parameter–property relationship, a homogeneous particle distribution in the polymer matrix yielding a reliable filament quality is essential.

## 1. Introduction

Nowadays, additive manufacturing, or often denoted as 3D printing with its manifold variants, is established for the fabrication of components of different sizes and technical functions covering the wide field of rapid prototyping and in certain cases rapid manufacturing of small-scale series [1,2,3,4,5,6,7,8]. According to DIN/ISOASTM 52900 [9], all 3D printing technologies can be attributed to the following classes or technologies:

(a) Binder Jetting (BJT);
(b) Directed Energy Deposition (DED);
(c) Material Extrusion (MEX);
(d) Material Jetting (MJT);
(e) Powder Bed Fusion (PBF);
(f) Sheet Lamination (SHL);
(g) Vat Photopolymerization (VPP).

Following the historical process development, the most relevant and robust technologies are Stereolithography (SLA) for photopolymerizable resins, Fused Filament Fabrication (FFF) or Fused Deposition Modeling (FDM) for polymer melts, and Selective Laser Sintering (SLS), Selective Laser Melting (SLM) or Electron Beam Melting (EBM) for polymer or metal powders. Initially, most of these methods were developed for only one material class, like SLA, only for pure organic reactive resins, but nowadays ceramic parts can be realized as well by SLA using ceramic filled resins applying the CeraFab process established by Lithoz [10]. A similar progress is shown in FFF printing: This polymer melt technology was originally developed only for thermoplastics by adapting the process characteristics from powder injection molding; a realization of small metal and ceramic parts is now established [11,12,13,14,15,16]. Nowadays, and according to DIN/ISOASTM 52900 [9], FFF and its variants should be denoted as material extrusion or shortly as MEX. Modern commercial MEX printers with a purchase price significantly below 5000 EUR possess a robust mechanical construction and a reproducible 3D printing quality for commercial standard thermoplastics, like polylactide (PLA) or acrylonitrile butadiene styrene (ABS) with printing temperatures below 300 °C. With respect to material development and usage of non-commercial materials, an open-source printing control software is mandatory for adjusting the individual printing parameters like built platform temperature as well as printing temperature and speed. MEX printing of high-performance thermoplastics, like polyetheretherketone (PEEK) and related materials, requires a more sophisticated printer setup and higher printing temperatures up to 500 °C, accompanied with a more complex printhead with, e.g., liquid cooling [17]. These polymers with their outstanding thermomechanical properties, like continuous operation temperature up to 300 °C under a low mechanical load or chemical resistivity against organic solvents even at elevated temperatures, have the potential for manifold applications substituting metals in chemical or process engineering, microfluidics, biomedical engineering or as mold insert material in polymer melt processing [18,19,20,21,22].

The introduction of functional properties to technical thermoplastics by the addition of inorganic nano- and micro-sized fillers, like carbon fibers (electrical conductivity, mechanical reinforcement), glass fibers (mechanical reinforcement), carbon black (coloring, electrical conductivity), and titania (coloring), was a well-established process for many decades [23,24]. Typically, compounding occurred by extrusion at moderate elevated temperatures depending on the applied polymer matrix. As an example, dielectric and magnetic properties were introduced into ABS (Acryl-Butadiene-Styrene) by using a filament extruder and subsequent printing [25,26]. The compounding process was performed separately prior to filament making in a mixer-kneader system guaranteeing a homogenous composite [25,26]. Sam–Daliri et al. investigated compounds consisting of glass fiber reinforced polypropylene (PP) waist and additional PP for proper glass fiber amount adjustment and the resulting mechanical properties [27]. They used for filament fabrication an almost identical extruder and filament maker like in [25,26]. Due to the PP matrix moderate compounding and filament making temperatures around 220 °C could be applied. Compounds made from Polyamid 12 (PA12), filled with MWCNT and copper fibers, could be realized by the usage of a twin-screw-extruder at 240 °C [L/D: 40) and then injection molded at 245 °C [28]. Polymer matrix composite (PMC) printing by MEX was established mostly using small amounts of fillers achieving additional functionalities or for decorative purposes. A lot of commercially available PMC-filaments are on market. Beyond the standard MEX polymers, like ABS, PLA, PETG, HIPS and others, high-performance polymers, like PEEK, PSU, PPS a.o. as well as derived PMC, are due to very few numbers of commercially available suitable MEX printers, and the difficulty of composite formation due to the very high processing temperatures. Therefore, in this work, the focus is set on the modification and characterization of the commercially available high-performance thermoplastic PEEK with fillers targeting property tailoring and printing parameter development for the realization of defect-free MEX-printed parts. Preliminary results were presented during the MicroSystemTechnik Kongress 2021 [22].

## 2. Materials and Methods

### 2.1. Material Selection and Composite Generation

Two different materials were used: first, commercially available PEEK and carbon fiber reinforced PEEK filaments were investigated; Table 1 shows the relevant material properties. It is obvious that the addition of carbon fibers causes a pronounced enhancement of the mechanical properties as well as of the thermal conductivity, which is favorable for, e.g., the use as mold insert material in polymer melt processing. In the second approach, different polymer matrix composites consisting of commercial PEEK (Victrex PEEK 450G, Victrex Europe GmbH, Hofheim, Germany) and different fillers, like Multiwall Carbon Nanotubes (CNT), copper powder and copper fibers targeting a property tailoring of the matrix, were used. The CNTs and the copper fibers are the same like the ones used in [28]. These composites were prepared applying a commercial single screw filament extruder (Filament Maker, 3Devo B.V., Utrecht, The Netherlands). Table 2 lists the filler’s relevant material properties.

Following the recommendations for the extrusion of pure Victrex PEEK 450G (extrusion temperature 380–395 °C, extrusion speed 5.7 rpm), different PMC filaments were extruded and winded on a filament spool, the compounding parameters are listed in Table 3. Prior to compounding, the polymer and the used filler were premixed manually in a beaker under ambient conditions and dried at an elevated temperature.

### 2.2. Printing Parameter Selection

**Figure 1:**
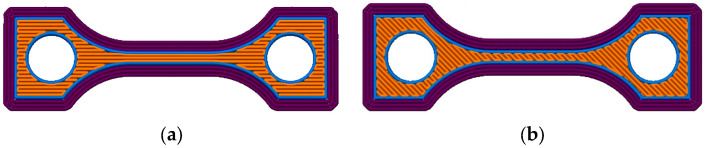
Schematic drawing of infill orientation during printing of each second layer in a tensile test specimen: (**a**) Parallel orientation (0°); (**b**) Tilted orientation (±45°) [22].

All test specimen and printing trials were performed applying the Apium P220 MEX-printer (Apium Additive Technologies GmbH, Karlsruhe, Germany). To investigate the influence of the printing direction on mechanical properties, the orientation of each second layer, relative to the previous one, was altered with a 0° or ±45° infill orientation angle (Figure 1). In all cases, a print-head nozzle diameter of 0.4 mm was applied. In addition, the printing temperature and the printing speed have a certain impact on the mechanical properties of the printed part due to the different melt viscosities at the printing nozzle and the melt fusion with the previously printed layer. Table 4 provides an overview about the printing parameter selection for the different commercial materials, as well as for the new composites. In all cases, the parameters were combined, e.g., at an extruder temperature of 485 °C at three different printing speeds were each tested with an infill orientation of 0° or 45°. The following notation was used: temperature/speed/infill orientation. As an example, 485/33.3/45 represents an extruder temperature (printing temperature) of 485 °C, a printing speed of 33.3 mm/s and an infill orientation of 45°. For the measurement of the thermal conductivity, test specimens of a different geometry were printed; optimized parameters are listed in Table 5.

### 2.3. Printed Sample Characterization

**Figure 2:**
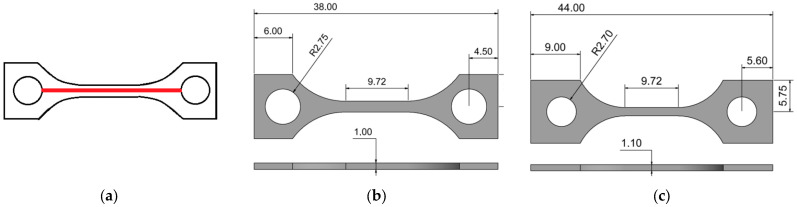
Specimen geometries (in mm): (**a**) Position of the measuring track (22 mm) for surface roughness detection; (**b**) PEEK and PEEK/copper samples, denoted as type A; (**c**). CF30 PEEK and PEEK/CNT samples, denoted as type B.

Three main aspects were investigated. First, the surface roughness was measured by a white light interferometer (MicroProf^®^ CWL F, FRT GmbH, Bergisch-Gladbach, Germany) according to the standard DIN EN ISO 4287 [29], investigating 10 (PEEK) or 5 (composites) samples each. The measured distance on the samples was 22 mm (Figure 2a); the used resolution was 1 µm with a sample rate of 32 Hz. Second, the tensile testing was performed according to DIN EN ISO 527-1 [30]. Applying a universal testing machine Z 100 (Zwick-Roell GmbH, Ulm, Germany), equipped with a 20 kN load cell and PMA 13/V7/1 (Maytec Mess- und Regeltechnik GmbH, Singen, Germany) extensometer. Two different specimen geometries, derived from DIN EN ISO 527-1 were used (Figure 2). A tensile specimen, derived from PEEK and PEEK/copper, followed the design shown in Figure 2b. Samples made from CF30 PEEK and PEEK/CNT used the slightly modified shape given in Figure 2c. The latter one was selected to avoid a fracture in the sample head area by increasing the mechanical stability of this region. After tensile testing, the fracture analysis was performed via SEM (Zeiss Gemini, Zeiss Microscopy GmbH, Oberkochen, Germany), To increase the conductivity, the fracture area was sputtered with gold (Emitech K575, Quorum Technologies Ltd., Laughton, UK). Third, the thermal conductivity was investigated by a combined method approach. The heat capacity was measured via DSC (dynamic scanning calorimetry, heating/cooling rate 10 K/min, −10–200 °C, argon atmosphere, three runs) applying a Netzsch DSC 204 (Netzsch Gerätebau, Selb, Germany) with sapphire as reference. The heat transfer was estimated by the laser-flash method (range 25–180 °C, 5 measurements) using a Netzsch LFA 427 (Netzsch Gerätebau, Selb, Germany).

## 3. Results and Discussion

### 3.1. Composite Formation and Filament Extrusion

**Figure 3:**
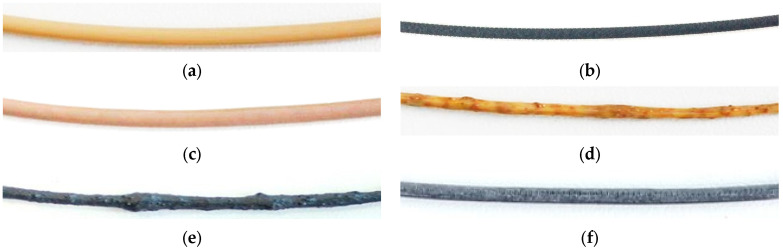
Images of extruded filaments (length 5 cm): (**a**) PEEK; (**b**) CF30 PEEK; (**c**) PEEK with 1.6 Vol% copper particles; (**d**) PEEK with 1.6 Vol% copper fibers; (**e**) PEEK with 2.3 Vol% CNT as extruded; (**f**) PEEK with 2.3 Vol% CNT postprocessed (grinded surface).

The two commercial filaments—PEEK and CF30 PEEK—could be extruded in a reliable quality and constant diameter (1.75 ± 0.05 mm) (Figure 3a,b) and could be used in MEX printing directly without any further postprocessing. The addition of copper particles to PEEK applying the 3Devo filament extruder yielded in a usable filament with a diameter of 1.75 ± 0.15 mm and a smooth surface (Figure 3c). The addition of fibers (copper and CNT) delivered non-homogenous filaments with variable diameter and very rough surfaces that hamper material flow during printing and generating pores during filament deposition. (Figure 3d,e). The copper fiber containing filament could therefore not be considered for further processing, whilst some filament sections of the CNT containing PEEK after surface grinding (Figure 3f) could be used for sample printing.

In all composite systems, the compounding capability in the 3Devo filament maker was not sufficient enough for reliable filament preparation. For further investigations, a pre-compounding in a commercial extruder seems to be mandatory. Previous work with ABS as matrix and ceramic nanoparticles described a poor filament quality according to insufficient particle deagglomeration and wetting during the compounding process, as well as applying a commercial mixer-kneader system [26].

### 3.2. MEX Printing of Tensile Test Specimen

**Figure 4:**
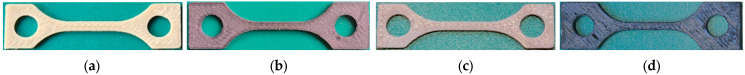
Images of printed tensile test specimen: (**a**) PEEK (type A); (**b**) CF30 PEEK (type B); (**c**) PEEK with 1.6 Vol% copper particles (type A); (**d**) PEEK with 2.3 Vol% CNT (type B).

Under consideration of the printing parameters for the different materials described in Table 4, tensile test specimens were printed considering the two different geometries shown in Figure 2. Exemplarily printed samples are shown in Figure 4.

### 3.3. Surface Roughness of the MEX Printed Samples

#### 3.3.1. Commercially Available Filaments: PEEK and CF30 PEEK

With respect to an acceptable experimental effort, only samples with a 45° printing orientation (Figure 1b) were investigated. With respect to the huge experimental error up to 20%, a clear correlation between printing speed and the surface roughness could not be found, hence, only the printing temperature was considered in the following. Table 6 lists for PEEK and Table 7 for CF 30 PEEK the different roughness values Ra, Rz and Rmax. The development of the different surface roughness values with printing temperature is not unique for PEEK and CF30 PEEK, while in the latter case, all measured values drop with increasing surface temperatures (Table 7); the related value for PEEK increases first from 420 °C to 440 °C and decreases with further temperature rising. This may be attributed to the relatively low first-printing temperature accompanied with a huge melt viscosity avoiding a surface planarization after printing, which can be overcome at elevated temperatures. The direct comparison of PEEK and CF30 PEEK, printed at 485 °C, delivers the composite material’s smaller roughness values. This may be attributed to the known suppression of the crystalline domains by the addition of micro-sized fillers promoting a higher amount of the amorphous domains with its smoother surface after solidification.

#### 3.3.2. New PEEK-Based Composites

Following the previous shown results, only specimens printed at the highest temperatures were investigated to obtain the smallest surface roughness values. Table 8 provides an overview about the measured data for better comparison; the smallest values for the two commercial filaments were added as well. All samples were printed with a 45° orientation. The different roughness values Ra, Rz and Rmax behave in a non-unique manner. Following only Rmax, all filled PEEK composites show smaller values than the pure printed PEEK. But, in general, a clear correlation between applied filler and resulting surface roughness cannot be made.

### 3.4. Mechanical Characterization

#### 3.4.1. Influence of the Printing Orientation on Tensile Strength

**Figure 5:**
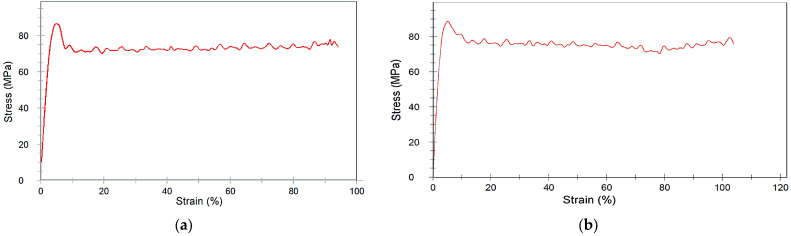
Stress–strain-curves of pure PEEK printed with different orientations; (**a**) parameter set 485/33/0; (**b**) parameter set 485/33/45.

As described in the literature, the printing orientation may have an influence on the resulting mechanical properties. Exemplarily, Figure 5 shows, for the two systems 485/33/0 and 485/33/45, the measured stress–strain-diagrams. Both diagrams are almost identical; the printed specimen with the 45° orientation is slightly more ductile. For all the obtained measured data, here, the tensile strength and Youngs modulus of the 0° and 45° orientation are compared in Table 9 for pure PEEK, and in Table 10 for the CF30 PEEK at different printing temperatures and speed. Regarding PEEK, the measured tensile strength of the samples with 45° orientation are slightly higher as the related ones with 0° orientation, but within the experimental error and, under identical printing conditions, the values are almost constant (Table 9). The values for the tensile strength and the Youngs modulus fit well with data taken from the literature (Table 1).

In contrast to pure PEEK, the presence of the carbon filler in CF30 PEEK caused a significant increase of the tensile strength, irrespective of the printing orientation (Table 10). The tensile strength values obtained at 0° orientation are—with one exception—higher than the ones with 45° orientation; the deviation ranges from 2–10% with an average value around 6%. The individual values should be not overestimated due to the given experimental error, but a clear trend can be observed. A positive effect on the Youngs modulus is shown in parallel printing orientation (0°) by an average enhancement around 19%. In a similar work, Zhen at al. investigated the impact of different printing parameters, like filling angle, extrusion rate and printing orientation (horizontal or vertical on the built platform), by applying a 0.4 mm nozzle and a constant printing temperature of 430 °C on the resulting mechanical properties [31]. Interestingly, they found for all investigated different extrusion rates, filling angles, and printing orientation smaller values for the tensile strength (max. 70 MPa). Unfortunately, the authors do not include neither the PEEKs vendor or data for tensile strength, taken from a data sheet [31]. Rahmatabadi and coworkers investigated the dependence of the mechanical properties with printing parameters like printing speed, printing orientation, and layer thickness a.o. of pure polyvinylchloride (PVC) [32]. They found that the orientation angle and the printing speed have a pronounced impact on the mechanical properties. But they also highlighted the significance of avoiding any voids or cavities in the printing part to enhance the mechanical properties [32].

#### 3.4.2. Influence of Printing Temperature and Speed on Tensile Strength

**Figure 6:**
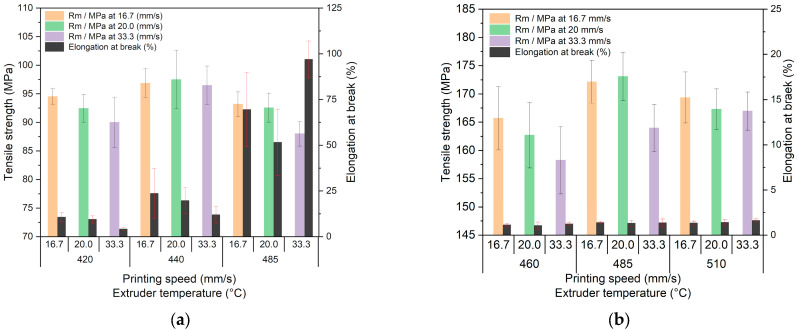
Impact of the printing parameters on mechanical properties; (**a**) PEEK; (**b**) CF30 PEEK.

Due to the small influence of the printing orientation on the tensile strength, the data are combined for a given set of printing parameters. For simplification, the measured values for the 45° and 0° orientations were averaged and combined. Figure 6a shows the change of the tensile strength with printing speed and temperatures for PEEK. Figure 6b shows the related data for CF30 PEEK. In case of PEEK, the Rm-value drops at constant temperature with printing speed within the frame of the experimental error. The same is valid for the elongation at break value at lower printing temperatures. Printing at 485 °C delivers, at all printing speeds, a significant elongation at break value, which can be interpreted as an induced ductility increase, which may be caused by the suppression of the crystalline phase (Figure 6a). The data sheet (see Table 1) lists for the used PEEK shows an elongation at the break value of 25% under DIN EN ISO 527-1 test conditions. The presence of carbon fibers enhances the tensile strength values, almost by a factor of 1.8.

As in pure PEEK, increasing printing speeds at a given temperature reduces the tensile strength of CF30 PEEK. The measured elongation at break values are significantly reduced to values below 2% when correlated with a more brittle behavior. The reference tensile strength value, provided by the supplier (see Table 1), is set to 190 MPa, and the elongation at the break value to 2%. Wang et al. measured, amongst others, the bending strength as a function of printing temperature, nozzle diameter and printing speed. The usage of the nozzle with 0.4 mm was very helpful for achieving the highest densities and bending strength at a extrusion temperature of 430 °C and 10 mm/s printing speed [20]. Applying an in-house built MEX-printer, Wang and coworkers investigated the impact of nozzle diameter, printing speed and printing temperature (380–440 °C) on surface roughness and tensile strength [33]. They reported, e.g., for the 0.4 mm nozzle, a tensile strength increase with increasing printing temperature. The found tensile strength values were around 70 MPa, as in [31]. Increasing printing temperatures caused a decrease of the surface roughness as well. Again, there was no information about the PEEKs vendor and a reference value for the tensile strength supported by the authors [33]. Clear correlations of tensile strength with printing speed, printing temperature and nozzle diameter could not be found [33], which agrees with the results presented here. Ding and coworkers researched, amongst others, the change of the tensile strength with printing temperature [34]. They used a commercial PEEK with a reference value of 109 MPa. In the printing temperature range from 360–420 °C, they described a non-uniform tensile strength behavior with a maximum value at 420 °C around 84 MPa, which is significantly lower than the reference value. The SEM fracture micrographs show a pronounced presence of voids in the samples [34]. An earlier study with another commercial PEEK (tensile strength reference value 100 MPa) yielded small tensile strength values below 60 MPa. Unfortunately, the authors did not support details about printing temperature and printing speed [35].

As mentioned in Section 3.1, the quality of the PEEK CNT composite filament was poor and needed grinding as a post-processing step. Consequently, a very few sample number could be realized, thus affecting an accurate and systematic investigation. Under consideration of three samples each, the impact of the printing orientation on the mechanical properties at a given temperature of 440 °C was the only investigation (Table 11). The presence of fibers can cause a property anisotropy when the PMC passes in the molten state through a nozzle with a small diameter, e.g., shown in injection molding of PA12/CNT composites [28]. As expected, the 0° printing orientation delivers a higher tensile strength. But both values are significantly smaller than for pure PEEK and CF30 PEEK. Any pronounced reinforcement cannot be observed, which should be attributed to the very poor filament properties (Figure 3e,f). Arif et al. examined the influence of increasing CNT content (1 and 3 wt%) on 3D printed PEEK-based composites [36]. They found a small decrease in the tensile strength relative to the pure PEEK (~4%), which is significantly smaller than the experimentally found value here using 6 wt% CNT (Table 11). Li et al. described the damage monitoring of thermoplastic laminates made from PA6, filled with 30 vol% carbon fibers using printed patches for defect repair when applying shear and tensile loading [37]. Doagou–Rad and coworkers investigated the impact of the same CNT (4 vol%) on the mechanical properties of PA12 [28]. Due to the enhanced compounding procedure using a dual-screw extruder, a homogenous PMC with increased tensile strength could be achieved. The addition of the CNTs led to a more brittle behavior [28].

**Figure 7:**
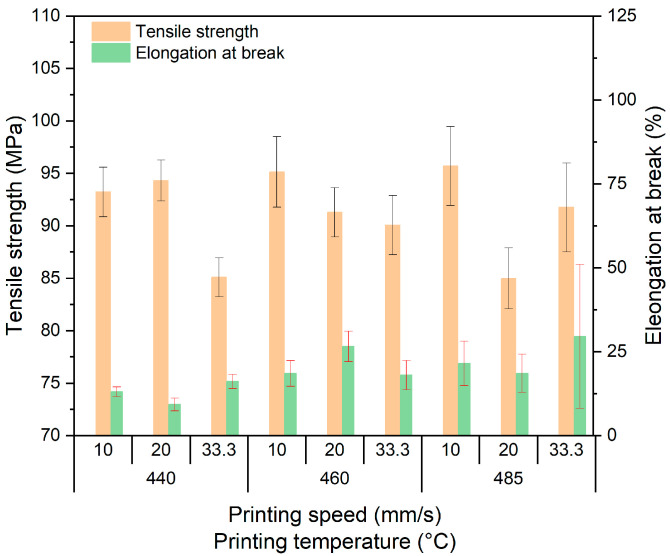
Impact of the printing parameters on mechanical properties of PEEK copper samples.

Due to the poor filament quality, only a reduced number of tensile test specimens, containing spherical copper particles, could be printed. Figure 7 shows the three investigated printing temperatures and printing speeds (all with 0° orientation) with the resulting mechanical properties. Whilst the printing temperature and speed have a non-systematic influence on the tensile strength, a trend for an increasing elongation at break with deposition temperature can be observed. In comparison with pure PEEK (Figure 6a), the tensile strength values are smaller up to 15%, depending on the printing parameters. A similar trend can be observed for the elongation at break values at low and middle printing temperatures.

#### 3.4.3. Fracture Imaging of PEEK

**Figure 8:**
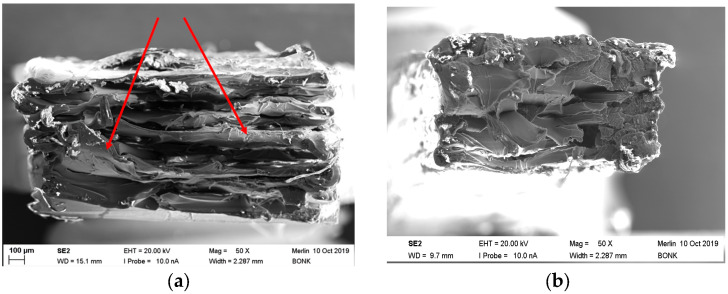
PEEK fracture images: (**a**) Parameter set: 440/16.7/45; (**b**) Parameter set: 485/33.3/45.

Often, MEX-printed samples suffer under non-optimized printing conditions from an insufficient adhesion between the different printed layers, causing under mechanical stress delamination. This can be attributed to an incomplete remelting of the previously printed polymer layer during printing of the new one, thus hampering a positive material joining. A printing temperature increase helps to reduce this drawback. Figure 8 shows, for two different PEEK printing temperatures, 440 °C (a) and 485 °C (b), fracture images after tensile testing. In case of the sample printed at 440 °C, massive defects can be detected. The specimen consists of individual separate layers with reduced interlayer adhesion and resulting delamination. The presence of layers is shown at the outer contours as well. Voids between the layers cannot be detected.

In addition, exfoliated layers (red arrows) can be observed (Figure 8a) also. In contrast, printing at 485 °C leads to a more compact and homogenous appearance without individual visible layers (Figure 8b). The higher processing temperature enabled a better melt fusion of the different layers. Interestingly, the tensile strength of the specimen printed at a lower temperature is slightly higher than the ones at larger printing temperatures. Due to the fact that the printing direction is mostly parallel to the applied mechanical load, delamination plays a minor role. In contrast, the elongation of break is significantly higher at the upper printing temperature, which can be interpretated as a change from a brittle to a ductile behavior (Figure 6a) by suppression of the crystalline domains.

#### 3.4.4. Fracture Imaging of PEEK-Based Composites

**Figure 9:**
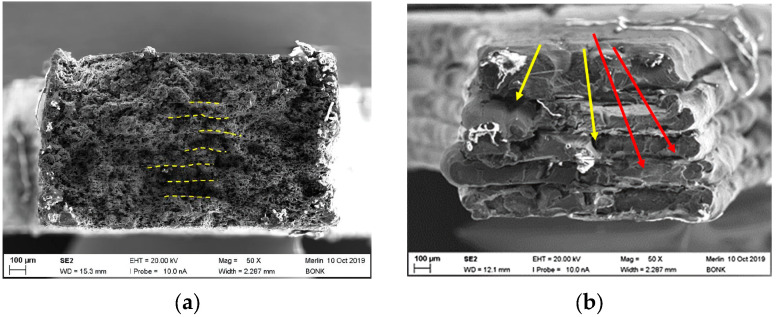
Fracture images: (**a**) CF30 PEEK, parameter set: 485/20/0; (**b**) PEEK CNT, parameter set: 440/10/0.

**Figure 10:**
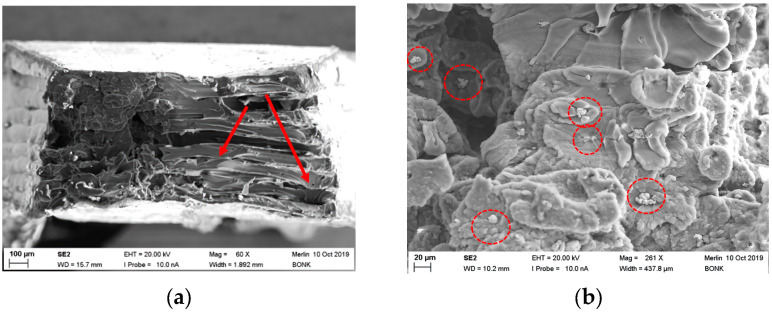
PEEK copper fracture images (parameter set: 460/10/0): (**a**) Overview; (**b**) Higher magnification for copper particle visualization.

As depicted from Figure 6b, the CF30 PEEK possesses its maximum tensile strength at a printing temperature of 485 °C and a printing speed of 20 mm/s; in all cases, a pronounced brittle behavior could be observed. Figure 9a shows the related fracture image. In contrast to the pure PEEK samples, a pronounced granular and rough surface is shown. A pull-out of the fibers out of the PEEK matrix cannot be observed, which can be interpretated as a good chemical coupling between the matrix and fiber, resulting in a reliable mechanical transfer of the applied tension to the high-resistant fiber. In the middle of the specimen, individual printed layers (dashed lines) without delamination under stress are shown. Due to the difficulties during filament compounding, only a very few number of tensile tests applying the new PEEK CNT composite could be performed. Figure 9b shows the fracture image of a sample printed at 440 °C. Individual layers of variable heights can be identified (red arrows), some voids (yellow arrows) between the layers are visible also. The fractured surface of the PEEK copper composite is presented in Figure 10 at different magnifications. In Figure 10a, the overview shows a lamellar appearance (red arrows) with delamination like the one seen from pure PEEK (Figure 8a). Small voids are located between the layers. A closer look at a higher magnification (Figure 10b) to the upper left section shows a different surface appearance. Via EDX, the small particles could be identified as copper particles (Figure 10b, red circles). It is apparent that either a phase separation occurred, or a poor compounding with insufficient homogenization and unwetted copper particles was presented, which is a more realistic outcome.

### 3.5. Thermal Conductivity

**Figure 11:**
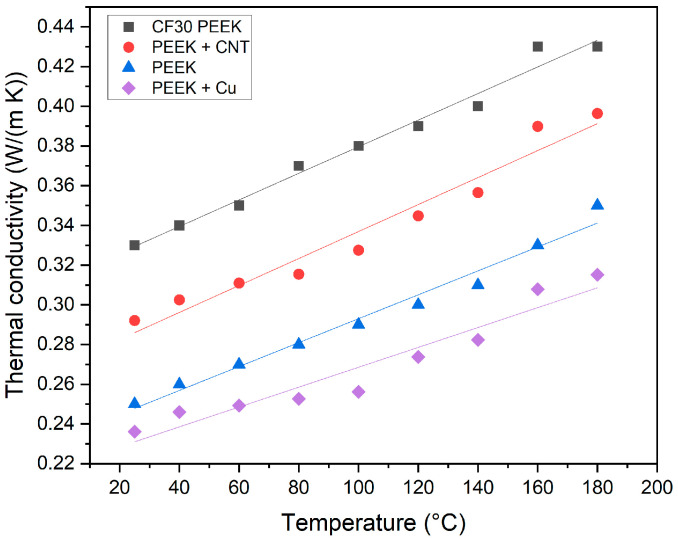
Temperature dependent thermal conductivity of all investigated samples.

All of the used fillers possess a higher thermal conductivity than the pure PEEK (Table 1 and Table 2). With respect to the usage of PEEK-based matrix composites, e.g., as mold insert material, the thermal conductivity must be significantly enhanced due to the essential mold insert tempering during the injection molding cycle, enabling successful molding and demolding. Figure 11 shows, for the investigated materials, the change of the thermal conductivity with temperature. Due to the test machine’s requirements, suitable specimens with optimized printing parameters were printed (Table 8).

Whilst the thermal conductivity value of PEEK, supplied by the vendor, could be confirmed, the related value for the commercial CF30 PEEK lies significantly lower than the information provided by the data sheet. In all cases, an increase of the thermal conductivity with temperature can be observed. Surprisingly, the addition of copper lowered the thermal conductivity, which must be attributed to the presence of voids in the sample, which is shown in the fracture image as well (Figure 10a). In case of the composites, the relatively small thermal conductivity values can be also attributed to the insufficient compounding process preventing any homogeneous particle distribution and the formation of thermal conductivity pathways. Doagou–Rad et al. investigated PMCs consisting of PA12 as polymer matrix and the same CNT as filler (4 vol%) in injection molding [28]. They also found only a small increase in the thermal conductivity (0.42 W/(m K) @25 °C) relative to the value for PA12 (0.37 W/(m K) @25 °C). Due to the fact that the sum thermal conductivity correlates with the volume fraction and the structural aspect ratio of the added filler in a PMC [28,38,39,40], the observed thermal conductance values are small. For certain applications, larger values are needed, as described in [28,39,40]. This seems to be challenging, according to the difficulties during compounding even of the given small filler amounts and the present bulk defects. But the results in [28] showed that, even at a solid load of 30 vol%, copper fibers delivered a small thermal conductivity value around 1.38 W/(m K) @25 °C.

## 4. Conclusions and Outlook

The main purpose of this work was the development of a new printable PMC with PEEK as the thermoplastic matrix and different fillers with good thermal conductivity. The usage of PEEK is a challenge due to the very high printing temperature beyond 400 °C, according to the huge melting temperature of almost 350 °C, which hampers the composite formation as well. The main knowledge gained and achievements of this work are:

- The combined compounding and filament fabrication suffered from the poor mixing and compounding capability of the used commercial equipment, delivering inhomogeneous filaments, poor filler wetting and a reduced printability.
- The addition of fillers like CNT and copper particles in combination with incomplete compounding may cause a poorer filament quality. A filament postprocessing, like surface grinding, can improve the filament quality.
- Increasing printing temperatures are favorable for smoother part surfaces and a more ductile behavior.
- The measured tensile strength of printed samples made out the two commercial filaments were close to the bulk values supported by the vendor. This is a strong hint for the absence of voids in the printed specimen and the provided optimized printing parameters.
- It is possible to enhance the thermal conductivity by the addition of suitable fillers, in this case, CNT. The generation of voids in the printed samples must be strictly avoided.

Future work should concentrate on an improved compounding procedure with better mixing and improved wetting of the particles by the polymer melt at temperatures beyond 410 °C, guaranteeing a low-melt viscosity. Then, larger filler amounts should be possible.

## Figures and Tables

**Table 1:** Material properties of used commercial PEEK filaments.

ITEM	PEEK ^1^	CF30 PEEK ^2^
Filament vendor	Apium	Ensinger
Type	450 natural, identical with Ensinger Tecafil VX natural 450	Tecapeek CF30 black
Glass transition temperature (°C)	143	143
Melting temperature (°C)	343	343
Young´s modulus (GPa)	3.6	17.5
Tensile strength (MPa)	100	190
Elongation at break (%)	25	2
Thermal conductivity (W/(m K))	0.25	0.66

^1^ Vendors data sheet: https://www.ensingerplastics.com/en/filaments/tecafil-peek-vx-natural-1-75-mm#/product-technical-detail-collapse-item-4-lvl-1; ^2^: Vendors data sheet: https://www.ensingerplastics.com/en/filaments/tecafil-peek-vx-cf30-black-1-75-mm#/product-technical-detail-collapse-item-1-lvl-1, both accessed on 26 June 2023.

**Table 2:** Material properties of used fillers for composite formation.

ITEM	CNT ^1^	Copper Powder ^1^	Copper Fibers ^1^
Vendor	Nanocyl S.A.	Atlantic Equipment Engineers	Deutsches Metallfaserwerk
Vendor’s affiliation	Sambreville, Belgium	Upper Saddle River, NJ, USA	Neidenstein, Germany
Type	NC7000	CU-110	STAX Cu99
Particle size	10.4 nm	8–11 µm	~60 µm
Fiber length	1.5 µm	n.a.	0.5–5 mm
Morphology	Fiber	Spherical	Fiber
Density (g/cm^3^)	1.7	8.9	8.9
Thermal conductivity (W/(m K))	3000	383	383

^1^ Material data taken from vendors data sheets.

**Table 3:** Compounding conditions using 3Devo Filament Maker.

ITEM	CNT	Copper Powder	Copper Fibers
Solid load (wt%)	6	10	10
Solid load (vol%)	2.3	1.6	1.6
Drying conditions	3 h, 150 °C	5 h, 120 °C	5 h, 120 °C
Extrusion temperature (°C)	385–405	390–410	390–410
Extrusion speed (rpm)	6	5.7	5.7

**Table 4:** Tensile test specimen printing parameters for all printable materials.

ITEM	PEEK	CF30 PEEK	PEEK Copper	PEEK CNT
Extruder temperature (°C)	485	510	485	440
	440	485	460	
	420	460	440	
Printing speed (mm/s)	33.3	33.3	33.3	10
	20	20	20	
	16.7	16.7	10	
Infill orientation (°)	0	0	0	0
	45	45		45

**Table 5:** Optimized printing parameters for thermal conductivity specimen.

Material	Extrusion Temperature (°C)	Printing Speed (mm/s)
PEEK	485	20
CF30 PEEK	510	20
PEEK/CNT	460	10
PEEK/copper	440	10

**Table 6:** Surface roughness of the printed PEEK samples as function of the printing temperature (in total 30 samples measured).

Printing Temperature (°C)	420	440	485
Ra (µm)	16	18	17
Rz (µm)	122	122	107
Rmax (µm)	141	152	139

**Table 7:** Surface roughness of the printed CF30 PEEK samples as function of the printing temperature (in total 15 samples measured).

Printing Temperature (°C)	460	485	510
Ra (µm)	10	9	7
Rz (µm)	69	65	56
Rmax (µm)	95	82	72

**Table 8:** Smallest surface roughness values of all investigated samples (number of measured samples in brackets).

Material (Sample Amount)	Ra (µm)	Rz (µm)	Rmax
PEEK (10)	17	107	139
CF30 PEEK (5)	7	56	72
PEEK CNT (3)	22	115	135
PEEK copper (3)	8	28	75

**Table 9:** Impact of the PEEK printing orientation on tensile strength and Youngs Modulus.

Printing Parameters	Tensile Strength (MPa) 0° Orientation	Youngs Modulus (Gpa) 0° Orientation	Tensile Strength (Mpa) 45° Orientation	Youngs Modulus (Gpa) 45° Orientation
420/16.7	91	3.1	95	3.4
420/20.0	91	4.3	94	3.6
420/33.3	87	3.6	93	3.5
440/16.7	95	3.5	99	3.6
440/20.0	97	3.9	98	3.6
440/33.3	95	3.8	98	3.5
485/16.7	94	3.5	96	3.0
485/20.0	91	3.2	94	3.4
485/33.3	87	3.0	89	4.0

**Table 10:** Impact of the CF30 PEEK printing orientation on tensile strength and Youngs modulus.

Printing Parameters	Tensile Strength (MPa) 0° Orientation	Youngs Modulus (GPa) 0° Orientation	Tensile Strength (MPa) 45° Orientation	Youngs Modulus (GPa) 45° Orientation
460/16.7	174	22	157	17
460/20.0	170	20	155	17
460/33.3	157	19	159	17
485/16.7	174	18	170	17
485/20.0	178	19	168	17
485/33.3	163	18	165	15
510/16.7	172	19	167	18
510/20.0	173	20	161	16
510/33.3	170	23	164	16

**Table 11:** Mechanical properties of the investigated PEEK CNT system.

PEEK CNT Parameter Set	Tensile Strength (MPa)	Elongation at Break (%)
440/10/0	75.0 ± 2.8	2.0 ± 0.7
440/10/45	69.2 ± 2.7	1.5 ± 0.3

## Data Availability

Not applicable.
